# Prevalence and treatment of atherogenic dyslipidemia in the primary prevention of cardiovascular disease in Europe: EURIKA, a cross-sectional observational study

**DOI:** 10.1186/s12872-017-0591-5

**Published:** 2017-06-17

**Authors:** Julian P. Halcox, José R. Banegas, Carine Roy, Jean Dallongeville, Guy De Backer, Eliseo Guallar, Joep Perk, David Hajage, Karin M. Henriksson, Claudio Borghi

**Affiliations:** 1 Institute of Life Sciences 2, Swansea University College of Medicine, Singleton Park, Swansea, SA2 8PP UK; 2 Department of Preventive Medicine and Public Health, School of Medicine, Universidad Autónoma de Madrid/IdiPaz and CIBER of Epidemiology and Public Health (CIBERESP), Madrid, Spain; 3 INSERM CIC-EC 1425 and Département d’Épidémiologie et Recherche Clinique, Assistance Publique–Hôpitaux de Paris, Hôpital Bichat, Paris, France; 4 INSERM U 744, Institut Pasteur de Lille, Université Lille-Nord de France, Lille, France; 5 Department of Public Health, University of Ghent, Ghent, Belgium; 6 Departments of Epidemiology and Medicine and Welch Center of Prevention, Epidemiology and Clinical Research, Johns Hopkins Bloomberg School of Public Health, Baltimore, MD USA; 7 School of Health and Caring Sciences, Linnaeus University, Kalmar, Sweden; 8 Department of Medical Sciences, Uppsala University, Uppsala, Sweden; 9 Department of Internal Medicine, Ageing and Clinical Nephrology, University of Bologna, Bologna, Italy

**Keywords:** Atherogenic dyslipidemia, Cardiovascular disease, Epidemiology, Risk factors/global assessment

## Abstract

**Background:**

Atherogenic dyslipidemia is associated with poor cardiovascular outcomes, yet markers of this condition are often ignored in clinical practice. Here, we address a clear evidence gap by assessing the prevalence and treatment of two markers of atherogenic dyslipidemia: elevated triglyceride levels and low levels of high-density lipoprotein cholesterol.

**Methods:**

This cross-sectional observational study assessed the prevalence of two atherogenic dyslipidemia markers, high triglyceride levels and low high-density lipoprotein cholesterol levels, in the study population from the European Study on Cardiovascular Risk Prevention and Management in Usual Daily Practice (EURIKA; *N =* 7641; of whom 51.6% were female and 95.6% were White/Caucasian). The EURIKA population included European patients, aged at least 50 years with at least one cardiovascular risk factor but no history of cardiovascular disease.

**Results:**

Over 20% of patients from the EURIKA population have either triglyceride or high-density lipoprotein cholesterol levels characteristic of atherogenic dyslipidemia. Furthermore, the proportions of patients with one of these markers were higher in subpopulations with type 2 diabetes mellitus or those already calculated to be at high risk of cardiovascular disease. Approximately 55% of the EURIKA population who have markers of atherogenic dyslipidemia are not receiving lipid-lowering therapy.

**Conclusions:**

A considerable proportion of patients with at least one major cardiovascular risk factor in the primary cardiovascular disease prevention setting have markers of atherogenic dyslipidemia. The majority of these patients are not receiving optimal treatment, as specified in international guidelines, and thus their risk of developing cardiovascular disease is possibly underestimated.

**Trial registration:**

The present study is registered with ClinicalTrials.gov (ID: NCT00882336).

**Electronic supplementary material:**

The online version of this article (doi:10.1186/s12872-017-0591-5) contains supplementary material, which is available to authorized users.

## Background

Although considerable progress has been made in understanding and treating associated risk factors, cardiovascular disease (CVD) remains the leading cause of death among adults under the age of 75 years in Europe [1]. Traditionally recognized risk factors for CVD include age, male sex, smoking, lack of exercise, obesity, hypertension, high levels of low-density lipoprotein cholesterol (LDL-C), type 2 diabetes mellitus (T2DM), and familial predisposition [2]. In addition, other factors, including atherogenic dyslipidemia, and elevated lipoprotein(a) or C-reactive protein (CRP) levels, may also be important considerations when estimating patients’ overall CVD risk [2–9]. Atherogenic dyslipidemia has been characterized as a combination of elevated LDL-C and triglyceride (TG) levels, decreased high-density lipoprotein cholesterol (HDL-C) levels, and a preponderance of small-dense LDL-C particles [10]. Here, we use data from the European Study on Cardiovascular Risk Prevention and Management in Usual Daily Practice (EURIKA, ClinicalTrials.gov identifier: NCT00882336), a cross-sectional observational study including data on patients from 12 European countries with at least one traditional CVD risk factor but no history of cardiovascular events [11, 12], to assess the prevalence and treatment of two markers of atherogenic dyslipidemia: elevated TG levels and low HDL-C levels. We have previously assessed the prevalence of elevated levels of CRP in the EURIKA population [9]. Among patients without T2DM not receiving a statin, approximately half had CRP levels of at least 2 mg/l. The impact of CRP and markers of atherogenic dyslipidemia on CVD risk is often underestimated in clinical practice, with a lack of evidence for the prevalence and treatment of the latter. TG and CRP levels are not taken into account in global cardiovascular risk calculators, such as the Systematic Coronary Risk Evaluation (SCORE) algorithm [13] and the risk calculator developed alongside the American College of Cardiology/American Heart Association (ACC/AHA) 2013 guidelines [14].

## Methods

### Study design and participants

EURIKA was a cross-sectional study carried out in 12 European countries (Austria, Belgium, France, Germany, Greece, Norway, Russia, Spain, Sweden, Switzerland, Turkey, and the UK) [11]. Included patients were aged 50 years or older and had at least one risk factor for CVD but no history of cardiovascular events. Data collection started in May 2009 and ended in January 2010, with a 3-month data-collection period for each country. The study protocol was approved by the appropriate clinical research ethics committees in each participating country, and all patients provided signed informed consent. The methods for the study have been reported in detail elsewhere [12]. Briefly, the study sample was selected in a two-stage process that involved the random selection of both physicians and patients [12, 15]. In the first stage, primary care physicians (PCPs) and specialists involved in CVD prevention (including cardiologists, endocrinologists, and internal medicine specialists) were randomly selected for invitation to participate using the OneKey database (Cegedim Dendrite, Boulogne-Billancourt, France) [16]. In total, 809 physicians (approximately 60 per country) agreed to participate in EURIKA, 64% of whom were PCPs [15]. In the second stage, participating physicians sequentially invited patients who met the selection criteria (aged ≥50 years, who were free from CVD but with at least one major cardiovascular risk factor [dyslipidemia, hypertension, current smoker, T2DM, or obesity]). Hypertension was defined as having a systolic blood pressure (SBP) of at least 140 mmHg, a diastolic blood pressure (DBP) of at least 90 mmHg, or receiving treatment with antihypertensive medications. Approximately 600 patients were included per country, with a final sample size of 7641.

### Assessment of CVD risk factors

Demographic information and other details of participating patients were gathered from medical records and patient interviews. For each patient, a physical examination was conducted, blood pressure measured, and a 12-h fasting blood sample collected within 1 day of the initial outpatient consultation [12]. Blood pressure was measured under standardized conditions, and blood sample analysis was performed at a central laboratory (BioAnalytical Research Corporation, Ghent, Belgium), with the exception of patients in Russia (approximately 5% of the total patient population), for whom a laboratory analysis was performed locally. HDL-C concentration was measured using a modified enzymatic method, total cholesterol concentration using the cholesterol oxidase/p-aminophenazone (CHOD-PAP) method, TG concentration using the glycerol-3-phosphate-oxidase/p-aminophenazone (GPO-PAP) method, and CRP levels using a high-sensitivity immunoturbidimetric method (all using the Roche Modular P chemistry analyzer [Roche Diagnostics, Indianapolis, IN, USA]). LDL-C concentration was calculated by the Friedewald formula [17]. The ACC/AHA risk calculator was used to calculate 10-year CVD risk scores, and the version of the SCORE algorithm updated to consider patients’ total cholesterol and HDL-C levels as independent variables (SCORE-HDL) [6, 13, 14, 18]. Patients were considered to be at high CVD risk if they had a score of at least 7.5% when using the ACC/AHA risk calculator, or at least 5% when using the SCORE-HDL algorithm.

High TG levels were defined as those of at least 2.3 mmol/l (200 mg/dl), and low HDL-C levels as those lower than 1.0 mmol/l (40 mg/dl) in men and lower than 1.3 mmol/l (50 mg/dl) in women. For statin treatment, therapy was categorized as low or moderate intensity (pravastatin 5–40 mg/day, simvastatin 2.5–80 mg/day, lovastatin 10–80 mg/day, fluvastatin 10–80 mg/day, atorvastatin 5–40 mg/day, or rosuvastatin 5–20 mg/day), or high intensity (atorvastatin ≥40 mg/day or rosuvastatin ≥20 mg/day).

### Statistical analyses

Data are presented as mean and standard deviation for continuous variables, and as frequency and percentage for categorical variables. Comparisons between groups were performed using Student’s *t*-tests for normally distributed continuous variables, Mann–Whitney U tests for continuous variables that were not normally distributed, and χ^2^ or Fisher’s exact tests for categorical variables, as appropriate. A *p* value below 0.05 was considered significant. Multivariate logistic regression was performed to assess factors associated with high TG and/ or low HDL-C levels. Variables considered in the multivariate analysis included country of origin, age, sex, hypertension, obesity, T2DM status, smoking status, total cholesterol levels, CRP levels, use of β-blockers, use of α-adrenergic antagonists, and use of diuretics. A stepwise (bidirectional) selection method was used to keep only those variables statistically significantly associated at the *p* < 0.05 level in the final model. Statistical analyses were performed using SAS version 9.2 (SAS Institute Inc., Cary, NC, USA).

## Results

### Overall patient characteristics

The EURIKA study population consisted of 7641 patients with a mean age of 63.2 years, of whom 48.4% were men and of whom 95.6% were White/Caucasian (Table 1). Mean body mass index (BMI) was 28.9 kg/m^2^, 21.0% of patients were current smokers, and 26.8% of participants had T2DM. Mean alcohol consumption of the EURIKA study population was 5.7 units/week, and 19.8% of patients reported undertaking no physical exercise. Almost three-quarters (72.8%) of patients had hypertension.

**Table 1:** Patient characteristics and treatment

	Overall (*N* = 7641)	High TG (*n* = 1591)	Low HDL-C (*n* = 1691)	High TG and low HDL-C (*n* = 759)
Age (year)	63.2 (9.0)	61.5 (8.4)	62.0 (8.8)	60.8 (8.5)
Sex				
Male (*n,* %)	3696 (48.4)	903 (56.8)	623 (36.8)	330 (43.5)
Female (*n,* %)	3945 (51.6)	688 (43.2)	1068 (63.2)	429 (56.5)
Race				
White/Caucasian (*n*, %)	6675 (95.6)	1408 (94.9)	1515 (94.3)	681 (94.7)
Middle East/North African (*n*, %)	74 (1.1)	15 (1.01)	23 (1.4)	9 (1.3)
South Asian (*n*, %)	63 (0.9)	16 (1.01)	21 (1.3)	6 (0.8)
Other Asian countries (*n*, %)	34 (0.5)	10 (0.7)	8 (0.5)	2 (0.3)
South American origin (*n*, %)	22 (0.3)	6 (0.4)	8 (0.5)	4 (0.6)
Sub-Saharan (*n*, %)	15 (0.2)	1 (0.1)	2 (0.1)	1 (0.1)
Caribbean (*n*, %)	14 (0.2)	2 (0.1)	2 (0.1)	1 (0.1)
Other (*n*, %)	82 (1.2)	26 (1.8)	28 (1.7)	15 (2.1)
Current smoker (*n,* %)	1608 (21.0)	393 (24.7)	397 (23.5)	201 (26.5)
BMI (kg/m^2^)	28.9 (5.5)	30.6 (5.4)	30.9 (5.8)	31.3 (5.4)
≥ 30 kg/m^2^ (*n,* %)	2788 (37.0)	783 (49.6)	858 (50.7)	410 (54.0)
T2DM^a^ (*n,* %)	2046 (26.8)	562 (35.3)	617 (36.5)	305 (40.2)
Blood pressure				
Hypertension^b^ (*n,* %)	5559 (72.8)	1193 (75.2)	1278 (75.6)	564 (74.3)
SBP (mmHg)	135.1 (16.6)	136.9 (16.7)	135.5 (17.1)	136.0 (17.0)
DBP (mmHg)	80.9 (9.9)	82.3 (10.1)	81.7 (10.0)	82.5 (9.9)
Serum lipid levels				
TC (mmol/l)	5.5 (1.1)	5.9 (1.2)	5.2 (1.2)	5.7 (1.2)
LDL-C (mmol/l)	3.2 (1.0)	3.3 (1.1)	3.1 (1.0)	3.2 (1.0)
Non-HDL-C (mmol/l)	4.0 (1.1)	4.7 (1.2)	4.2 (1.2)	4.7 (1.2)
TG (mmol/l)	1.8 (1.3)	3.4 (1.9)	2.6 (2.0)	3.8 (2.5)
HDL-C (mmol/l)	1.4 (0.4)	1.2 (0.3)	1.0 (0.2)	1.0 (0.2)
CRP (mg/l)	4.2 (8.7)	4.4 (6.3)	6.0 (12.2)	5.0 (7.5)
LLT				
At least one LLT (*n,* %)	3278 (42.9)	757 (47.6)	749 (44.3)	349 (46.0)
Statin alone (*n,* %)	2862 (37.5)	598 (37.6)	617 (36.5)	258 (34.0)
Statin + other LLT (*n,* %)	178 (2.3)	74 (4.7)	57 (3.4)	43 (5.7)
Other LLT alone (*n,* %)	238 (3.1)	85 (5.3)	75 (4.4)	48 (6.3)
Ezetimibe (*n,* %)	151 (2.0)	57 (3.6)	45 (2.7)	35 (4.6)
Fibrate (*n,* %)	220 (2.9)	84 (5.3)	68 (4.0)	45 (5.9)
Nicotinic acid (*n,* %)	6 (0.1)	3 (0.2)	4 (0.2)	3 (0.4)
Alcohol consumption^c^				
(units/week)	5.7 (11.3)	6.4 (12.2)	3.2 (6.4)	3.6 (6.8)
> 14 units/week (male; *n*, %)	529 (17.8)	129 (18.0)	47 (9.6)	27 (10.3)
> 7 units/week (female; *n*, %)	272 (8.9)	49 (9.4)	43 (5.5)	22 (6.9)
Physical exercise				
No exercise (*n*, %)	1489 (19.8)	368 (23.5)	406 (24.3)	191 (25.5)
Only light physical activity per week (*n*, %)	3782 (50.2)	807 (51.5)	910 (54.6)	403 (53.8)
Heavy physical activity 1–2 times per week (*n*, %)	1232 (16.4)	220 (14.1)	203 (12.2)	90 (12.0)
Heavy physical activity ≥3 times per week (*n*, %)	1026 (13.6)	171 (10.9)	149 (8.9)	65 (8.7)

Data are mean (standard deviation) unless otherwise indicated

High TG: ≥ 2.3 mmol/l. Low HDL-C: < 1.0 mmol/l in men and <1.3 mmol/l in women

^a^Considered present if the diagnosis was documented in the medical records

^b^SBP ≥ 140 mmHg, DBP ≥ 90 mmHg, or receiving antihypertensive medication

^c^Moderate alcohol consumption of 14 units/week (male) and 7 units/week (female) is outlined in the 2016 ESC/EAS guidelines for management of dyslipidemias [3]

*Abbreviations: BMI* body mass index, *CRP* C-reactive protein, *DBP* diastolic blood pressure, *HDL-C* high-density lipoprotein cholesterol, *LDL-C* low-density lipoprotein cholesterol, *LLT* lipid-lowering therapy, *SBP* systolic blood pressure, *T2DM* type 2 diabetes mellitus, *TC* total cholesterol, *TG* triglyceride

### Prevalence of high TG and/or low HDL-C levels

**Fig. 1:**
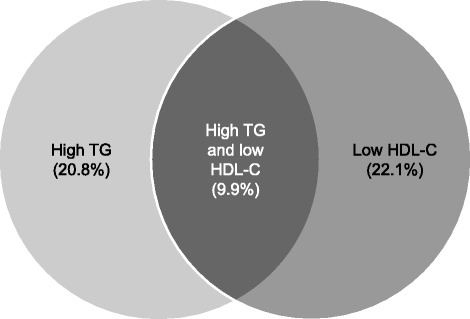
Prevalence of high TG and/or low HDL-C levels in the EURIKA population. Percentages indicated are of the total EURIKA population (*N* = 7641). High TG: ≥ 2.3 mmol/l. Low HDL-C: < 1.0 mmol/l in men and < 1.3 mmol/l in women *Abbreviations: EURIKA* European Study on Cardiovascular Risk Prevention and Management in Usual Daily Practice, *HDL-C* high-density lipoprotein cholesterol, *TG* triglyceride

Among the overall population that included treated and untreated patients, a total of 1591 patients (20.8%) were classified as having high TG levels (≥ 2.3 mmol/l), 1691 (22.1%) had low HDL-C levels (men: < 1.0 mmol/l; women: < 1.3 mmol/l), and 759 (9.9%) had both high TG and low HDL-C levels (Table 1; Fig. 1). A very small proportion of patients in the overall population had very high TG levels (> 5 mmol/L [1.9%]; > 10 mmol/L [0.3%]). Similarly, only 0.1% of the EURIKA population had very low HDL-C levels (< 0.5 mmol/L). The mean ages of patients in the subpopulations with high TG levels, low HDL-C levels, or both were similar to that of the overall population, as were mean SBP, DBP, and the proportions of patients with hypertension. There were higher proportions of patients classified as obese (BMI ≥ 30 kg/m^2^) in the subpopulations with high TG levels, low HDL-C levels, or both than in the overall population. There was a higher proportion of men with high TG levels than women in the patient population; conversely, the proportion of women was higher among patients with low HDL-C levels when compared with men. The proportion of patients who had T2DM was higher among patients with markers of atherogenic dyslipidemia than in the overall population. There were patients within the EURIKA population, both male and female, exceeding the recommended weekly limit of alcohol consumption. There were similar proportions of patients undertaking no physical exercise in the subpopulations with high TG levels, low HDL-C levels, or both to those in the overall population. We observed cross-country variation in the proportions of patients with markers of atherogenic dyslipidemia (Additional file 1: Table S1).

### Treatment of high TG and/or low HDL-C levels

**Fig. 2:**
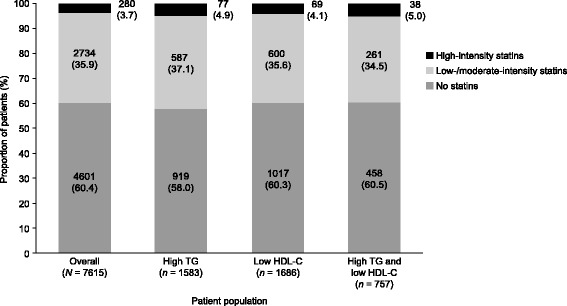
Proportion of patients treated with or without statins according to markers of atherogenic dyslipidemia. Data were missing for 26 patients in the overall population, 8 patients in the high TG group, 5 patients in the low HDL-C group, and 2 patients in the high TG and low HDL-C group. Data within bars are *n* (%). High TG: ≥ 2.3 mmol/l. Low HDL-C: < 1.0 mmol/l in men and < 1.3 mmol/l in women. *Abbreviations: HDL-C* high-density lipoprotein cholesterol, *TG* triglyceride

With regard to treatment, over half of patients in the overall population and in the subpopulations with markers of atherogenic dyslipidemia were not receiving any form of lipid-lowering therapy (LLT, Table 1). Of patients receiving LLT, most were receiving a statin alone. Only small proportions of patients were receiving ezetimibe, a fibrate, or nicotinic acid. Of those patients receiving a statin, most were receiving a low- or moderate-intensity statin; only a small proportion of patients (≤ 5%) were receiving a high-intensity statin (Fig. 2).

### Differences in the prevalence of high TG and/or low HDL-C levels according to statin treatment, T2DM, and CVD risk status

**Fig. 3:**
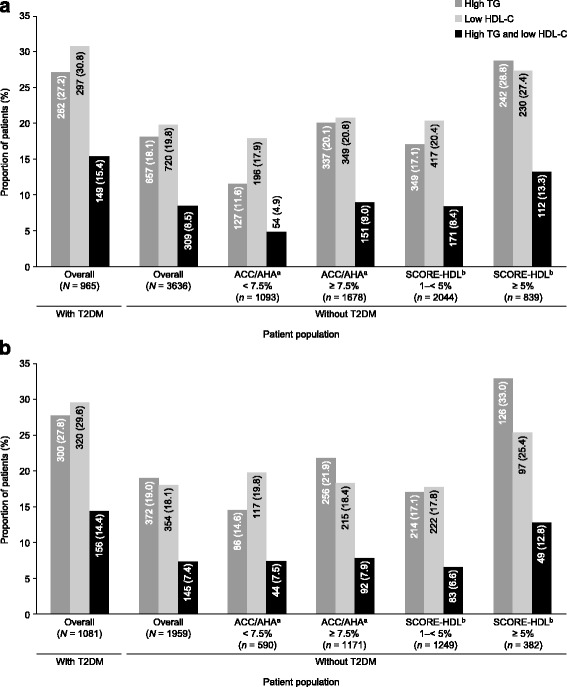
Proportion of patients with markers of atherogenic dyslipidemia, according to T2DM status and CVD risk. (**a**) Non-statin - treated patients; (**b**) statin-treated patients. Data within bars are *n* (%). High TG: ≥ 2.3 mmol/l. Low HDL-C: < 1.0 mmol/l in men and < 1.3 mmol/l in women. ^a^ACC/AHA risk calculator [14]. ^b^SCORE-HDL risk calculator [6, 18]. *Abbreviations: ACC* American College of Cardiology, *AHA* American Heart Association, *CVD* cardiovascular disease, *HDL-C* high-density lipoprotein cholesterol, *SCORE-HDL* Systematic Coronary Risk Evaluation-high-density lipoprotein, *T2DM* type 2 diabetes mellitus, *TG* triglyceride

Patients in the overall population were described according to whether or not they were receiving statin therapy, and then further stratified according to the presence or absence of T2DM, and then, among patients without T2DM, overall CVD risk assessed using either the ACC/AHA or SCORE-HDL risk calculators [6, 14]. Higher proportions of patients with T2DM were found to have high TG and/or low HDL-C levels than patients without T2DM (Fig. 3). Among patients without T2DM, greater proportions of patients in the higher overall CVD risk categories had high TG and/or low HDL-C levels than patients in lower overall CVD risk categories. These trends were observed in both statin-treated and non-statin-treated patients. Given that the SCORE-HDL and ACC/AHA risk calculators are valid only for assessing risk in routine clinical practice in patients up to 65 and 79 years of age, respectively, we went further in our analysis and stratified according to these age limits (Additional file 2: Figure S1 and Additional file 3: Figure S2). For the subgroups of patients up to the age of 65 years (SCORE-HDL) and 79 years (ACC/AHA), the proportions of patients with high TG and/or low HDL-C levels were similar to or slightly larger than the corresponding CVD risk groups for the whole population.

### Factors associated with high TG and/or low HDL-C levels

**Fig. 4:**
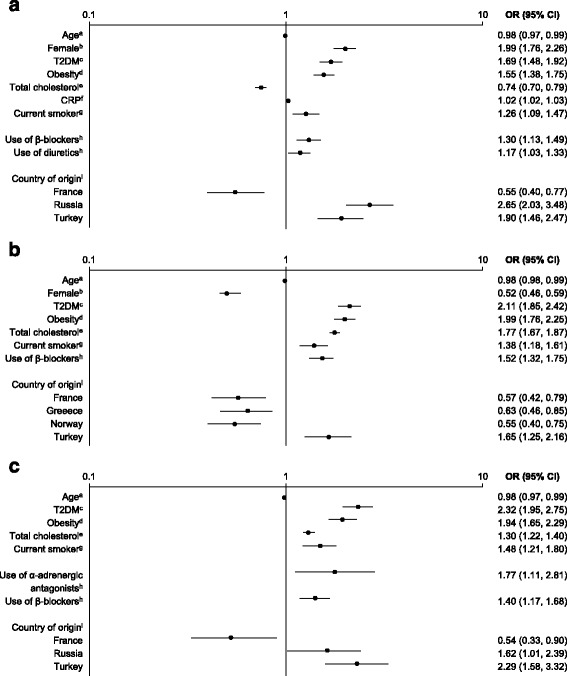
Multivariate analysis of factors associated with markers of atherogenic dyslipidemia. (**a**) Low HDL-C levels; (**b**) high TG levels; (**c**) low HDL-C and high TG levels. *p* < 0.0001 for all factors. Countries of origin with an OR that was not significant have been omitted. ^a^Per year. ^b^Relative to male participants. ^c^Relative to not having T2DM. ^d^BMI ≥ 30 kg/m^2^, relative to not being obese. ^e^Per mmol/l. ^f^Per mg/l. ^g^Relative to never smoking. ^h^Relative to non-use. ^i^Relative to the UK. *Abbreviations: BMI* body mass index, *CI* confidence interval, *CRP* C-reactive protein, *HDL-C* high-density lipoprotein cholesterol, *OR* odds ratio, *T2DM* type 2 diabetes mellitus, *TG* triglyceride

Factors significantly associated with high TG levels, low HDL-C levels, or both were assessed using multivariate analysis (*p* < 0.0001; Fig. 4). Female sex was positively associated with low HDL-C status but negatively associated with high TG levels. Other cardiovascular risk factors, including T2DM, obesity, smoking, and hypertension (indicated by the use of β-blockers) were positively associated with markers of atherogenic dyslipidemia. The association of markers of atherogenic dyslipidemia with CRP levels was found to be significant only after adjusting for confounding factors among patients with low HDL-C levels. Some variation in patients’ likelihood of having markers of atherogenic dyslipidemia was seen between countries of origin, when assessed relative to the UK.

### Association between high TG and/or low HDL-C levels and CRP

**Fig. 5:**
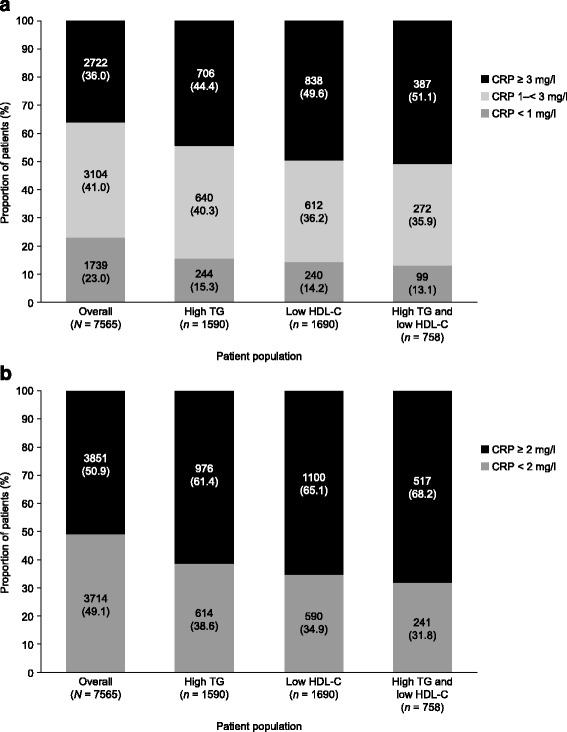
Association between markers of atherogenic dyslipidemia and CRP. (**a**) CRP levels of < 1 mg/L, 1–< 3 mg/L or ≥ 3 mg/L; (**b**) CRP levels < 2 mg/L or ≥ 2 mg/L. Data were missing for 76 patients in the overall population, and for 1 patient in each of the dyslipidemia groups**.** Data within bars are *n* (%)**.** High TG: ≥ 2.3 mmol/l. Low HDL-C: < 1.0 mmol/l in men and < 1.3 mmol/l in women. *Abbreviations: CRP* C-reactive protein, *HDL-C* high-density lipoprotein cholesterol, *TG* triglyceride

In the overall population, mean CRP was 4.2 mg/l, and this was increased in patients with high TG levels (4.4 mg/l), low HDL-C levels (6.0 mg/l), and both (5.0 mg/l) (Table 1). Patients in the overall population with high TG levels and/or low HDL-C levels were categorized by plasma CRP concentrations into two subpopulations: CRP lower than 1 mg/l, 1–< 3 mg/l, or at least 3 mg/l; and CRP lower than 2 mg/l or at least 2 mg/l. Greater proportions of patients had CRP levels of either at least 2 mg/l or at least 3 mg/l among those with high TG levels and/or low HDL-C levels than in the overall population, with between approximately 60 and 70% of patients with markers of atherogenic dyslipidemia having CRP levels of at least 2 mg/l, and between 45 and 50% having CRP levels of at least 3 mg/l (Fig. 5).

## Discussion

We have analyzed the number of patients with high levels of TG and/or low levels of HDL-C, two markers of atherogenic dyslipidemia, in the large clinical EURIKA population. We have shown that 20.8% of patients had high TG levels, 22.1% had low HDL-C levels, and 9.9% had both high TG and low HDL-C levels. Very few patients in our cohort had very high TG levels or very low HDL-C levels; it is likely that other comorbidities or underlying genetic factors may affect these individuals. Our analysis also reveals that the proportion of patients with T2DM (who are already considered to be at high risk of CVD) with high TG and/or low HDL-C levels is higher than that of patients without T2DM. In addition, when categorizing patients without T2DM according to the ACC/AHA or SCORE-HDL risk calculators, larger proportions of patients with high TG and/or low HDL-C levels are in the higher risk categories than in the lower risk categories. Cross-country variation in the proportions of patients with high TG and/or low HDL-C levels was observed and could be a consequence of genetic, cultural or socio-economic factors that have been discussed elsewhere [11]. We are unable to provide definitive explanations in the analysis presented here. Our multivariate analysis revealed that female sex was positively associated with low HDL-C status, which may reflect the specific selection criteria for the EURIKA population as females have higher HDL-C levels than males in the general population. Thus, our data suggest that European women with at least one cardiovascular risk factor but no history of cardiovascular disease are actually more likely than men to have a low HDL-C status.

The EURIKA study has provided important insights into the effectiveness of current practices related to primary CVD prevention in Europe [9, 11, 19]. The primary analysis of the EURIKA data demonstrated that a substantial proportion of patients had CVD risk factors that remained uncontrolled, despite receiving treatment [11]. A follow-up analysis of CRP levels in the EURIKA population revealed that among patients without T2DM who were not receiving statin treatment, more than one-third had CRP levels of at least 3 mg/l, while almost half had CRP levels of at least 2 mg/l [9]. Here, our analysis of CRP levels demonstrates that a greater proportion of patients with high TG and/or low HDL-C levels also have low-grade inflammation, evident by elevated CRP levels, than in the overall at-risk population. These patients are likely to be at an even higher risk of CVD than those with either atherogenic dyslipidemia or elevated CRP levels alone.

We have previously reported on the proportions of patients in EURIKA who were not receiving any form of LLT and who had uncontrolled LDL-C levels [19]. Elevated LDL-C levels are among the primary causal risk factors for cardiovascular disease, and are a component of the atherogenic dyslipidemia profile [2]. Over one-third of patients defined as being at high risk of CVD in our previous analysis were not receiving any form of LLT [19]. Moreover, LDL-C levels were controlled in only 40% of these patients at high risk of CVD who were receiving LLT [19]. Findings from the Centralized Pan-Regional Surveys on the Undertreatment of Hypercholesterolemia (CEPHEUS) were similar; only 49.4% of patients achieved their recommended LDL-C levels [20]. A literature review from 2004 also reported a widespread failure in the attainment of recommended lipid levels in patients treated with LLT [21]. Similarly, in the current analysis, we observed that approximately 55% of patients with high TG levels, low HDL-C levels, or both were not taking any form of LLT. Furthermore, of those patients treated with statins, the majority were using low-intensity statins. These observations build on our previous arguments that there is a clear opportunity to improve rates of treatment for primary CVD prevention, and for patients with dyslipidemia in particular.

Whether or not TG levels are a causal risk factor for CVD is debated [22, 23]; patients with high TG levels often have additional CVD risk factors; TG levels in human plasma are highly variable and are strongly associated with low HDL-C levels, which makes it difficult to separate the contributions of these two components [22–25]. Nevertheless, several population studies and meta-analyses have shown a significant link between TG levels and CVD risk, independent of other CVD risk factors, including HDL-C levels [26–28]. A topic of debate has been whether measuring non-fasting TG levels is a better predictor of CVD than measuring fasting TG levels, with some studies showing a stronger association between non-fasting TG levels and CVD risk than fasting TG levels [24, 29]. European guidelines have previously recommended the measurement of fasting TG levels, as was done in EURIKA, owing to a lack of standardization of non-fasting TG measurements [30]. Guidelines recommend lifestyle interventions in patients with moderately elevated TG levels (e.g. reduction in alcohol consumption, dietary modifications, or increased aerobic exercise) [23]. A reduction in alcohol consumption is of particular importance, as patients with elevated TG levels are likely to experience further increases from consuming even small quantities of alcohol [3]. Our analysis reveals that within the EURIKA population, small proportions of patients with elevated TG levels are consuming more units of alcohol than the recommended weekly limit. Furthermore, almost 20% of the overall population reported undertaking no physical exercise. In patients with TG levels above 500 mg/dl, pharmacological intervention in the form of fibrates, niacins, or omega-3 fish oils should be considered [23].

The EURIKA study has the strength of allowing analysis of data from a large sample of patients from multiple European countries according to standardized procedures. Almost all blood samples were analyzed at the same location, with the exception of patients from Russia. A limitation of the study, however, is that it is cross-sectional, and therefore does not allow conclusions to be drawn regarding the longitudinal association of individual factors with CVD risk. The EURIKA study recruited individuals over 50 years of age with at least one major CVD risk factor, including dyslipidemia. Therefore, the proportion of patients identified with low HDL-C and high TG levels is likely to be greater than in people of the same age in the general population. Previous cohort studies investigating the predictive power of HDL-C levels in CVD risk have measured HDL-C using precipitation methods. In EURIKA, HDL-C levels were measured using an enzymatic method; it has been demonstrated that enzymatic methods result in higher recorded HDL-C levels than precipitation methods [31]. Therefore, it is possible that we have underestimated the proportion of the cohort with low HDL-C levels. The participation rate among invited physicians was also low; however, potential patient selection bias is likely to have been reduced by the high participation rate among invited patients, and the randomized method of patient selection. Finally, we did not calculate the ratio of TG to HDL-C concentrations in the EURIKA study; however, this marker has been proposed to correlate with insulin resistance and thus could be used to identify patients at risk of CVD [32].

## Conclusions

A considerable proportion of primary prevention patients at risk of CVD in routine clinical practice have high levels of TG and low levels of HDL-C. Many of these patients also have evidence of elevated CRP levels, reflecting low-grade inflammation. Therefore, these patients’ absolute CVD risk may be underestimated by current European and US global risk calculators. These patients may benefit from more intensive or better-tailored treatment options to address their overall CVD risk, in accordance with evidence-based guidelines.

## Additional files

Additional file 1: Table S1. Prevalence of high TG and/or low HDL-C levels in the EURIKA population by country**.** Data are (*n,* %). High TG: ≥ 2.3 mmol/l. Low HDL-C: < 1.0 mmol/l in men and <1.3 mmol/l in women**.** *Abbreviations: HDL-C* high-density lipoprotein cholesterol, *TG* triglyceride (DOCX 15 kb)

Additional file 2: Figure S1. Proportion of non-statin-treated patients with markers of atherogenic dyslipidemia, according to T2DM status, CVD risk, and age: (a) according to SCORE-HDL categories; (b) according to ACC/AHA categories. Data within bars are *n* (%). High TG: ≥ 2.3 mmol/l. Low HDL-C: < 1.0 mmol/l in men and <1.3 mmol/l in women. *Abbreviations: ACC* American College of Cardiology, *AHA* American Heart Association, *CVD* cardiovascular disease, *HDL-C* high-density lipoprotein cholesterol, *SCORE-HDL* Systematic Coronary Risk Evaluation-high-density lipoprotein, *T2DM* type 2 diabetes mellitus, *TG* triglycerides (PDF 1099 kb)

Additional file 3: Figure S2. Proportion of statin-treated patients with markers of atherogenic dyslipidemia, according to T2DM status, CVD risk and age: (a) according to SCORE-HDL categories; (b) according to ACC/AHA categories. Data within bars are *n* (%). High TG: ≥ 2.3 mmol/l. Low HDL-C: < 1.0 mmol/l in men and <1.3 mmol/l in women. *Abbreviations: ACC* American College of Cardiology, *AHA* American Heart Association, *CVD* cardiovascular disease, *HDL-C* high-density lipoprotein cholesterol, *SCORE-HDL* Systematic Coronary Risk Evaluation-high-density lipoprotein, *T2DM* type 2 diabetes mellitus, *TG* triglycerides (PDF 1090 kb)

## Abbreviations

ACC: American College of Cardiology
AHA: American Heart Association
BMI: Body mass index
CEPHEUS: Centralized Pan-Regional Surveys on the Undertreatment of Hypercholesterolemia
CI: Confidence interval
CRP: C-reactive protein
CVD: Cardiovascular disease
DBP: Diastolic blood pressure
EURIKA: European Study on Cardiovascular Risk Prevention and Management in Usual Daily Practice
HDL-C: High-density lipoprotein cholesterol
LDL-C: Low-density lipoprotein cholesterol
LLT: Lipid-lowering therapy
OR: Odds ratio
PCP: Primary care physician
SBP: Systolic blood pressure
SCORE: Systematic Coronary Risk Evaluation
SCORE-HDL: Systematic Coronary Risk Evaluation-high-density lipoprotein
T2DM: Type 2 diabetes mellitus
TC: Total cholesterol
TG: Triglyceride
