# Supplementary material for: Prevalence and treatment of atherogenic dyslipidemia in the primary prevention of cardiovascular disease in Europe: EURIKA, a cross-sectional observational study
Source: BMC Cardiovasc Disord. 2017 Jun 17;17:160. doi: 10.1186/s12872-017-0591-5 (PMC5473961; doi:10.1186/s12872-017-0591-5)
Supplement: Supplementary file 1 — Prevalence of high TG and/or low HDL-C levels in the EURIKA population by country. Data are (n, %). High TG: ≥ 2.3 mmol/l. Low HDL-C: < 1.0 mmol/l in men and <1.3 mmol/l in women. Abbreviations: HDL-C high-density lipoprotein cholesterol, TG triglyceride (DOCX 15 kb) [file 12872_2017_591_MOESM1_ESM.docx]

|  | Overall  (*N* = 7641) | High TG  (*n* = 1591) | Low HDL-C  (*n* = 1691) | High TG and low HDL-C  (*n* = 759) |
| --- | --- | --- | --- | --- |
| Austria | 624 (8.2) | 152 (9.6) | 119 (7.0) | 62 (8.2) |
| Belgium | 638 (8.4) | 113 (7.1) | 133 (7.9) | 48 (6.3) |
| France | 593 (7.8) | 89 (5.6) | 64 (3.8) | 26 (3.4) |
| Germany | 678 (8.9) | 165 (10.4) | 143 (8.5) | 72 (9.5) |
| Greece | 620 (8.1) | 114 (7.2) | 115 (6.8) | 56 (7.4) |
| Norway | 611 (8.0) | 93 (5.9) | 132 (7.8) | 51 (6.7) |
| Russia | 604 (7.9) | 131 (8.2) | 252 (14.9) | 99 (13.0) |
| Spain | 642 (8.4) | 128 (8.1) | 114 (6.7) | 48 (6.3) |
| Sweden | 667 (8.7) | 112 (7.0) | 120 (7.1) | 58 (7.6) |
| Switzerland | 667 (8.7) | 156 (9.8) | 132 (7.8) | 71 (9.4) |
| Turkey | 663 (8.7) | 212 (13.3) | 227 (13.4) | 119 (15.7) |
| UK | 673 (8.8) | 126 (7.9) | 140 (8.3) | 49 (6.5) |

**Table S1** Prevalence of high TG and/or low HDL-C levels in the EURIKA population by country

Data are (*n,* %)

High TG: ≥ 2.3 mmol/l. Low HDL-C: < 1.0 mmol/l in men and < 1.3 mmol/l in women

*Abbreviations: HDL-C* high-density lipoprotein cholesterol, *TG* triglyceride
