# Supplementary material for: Prevalence and treatment of atherogenic dyslipidemia in the primary prevention of cardiovascular disease in Europe: EURIKA, a cross-sectional observational study
Source: BMC Cardiovasc Disord. 2017 Jun 17;17:160. doi: 10.1186/s12872-017-0591-5 (PMC5473961; doi:10.1186/s12872-017-0591-5)
Supplement: Supplementary file 2 — Proportion of non-statin-treated patients with markers of atherogenic dyslipidemia, according to T2DM status, CVD risk, and age: (a) according to SCORE-HDL categories; (b) according to ACC/AHA categories. Data within bars are n (%). High TG: ≥ 2.3 mmol/l. Low HDL-C: < 1.0 mmol/l in men and <1.3 mmol/l in women. Abbreviations: ACC American College of Cardiology, AHA American Heart Association, CVD cardiovascular disease, HDL-C high-density lipoprotein cholesterol, SCORE-HDL Systematic Coronary Risk Evaluation-high-density lipoprotein, T2DM type 2 diabetes mellitus, TG triglycerides (PDF 1099 kb) [file 12872_2017_591_MOESM2_ESM.pdf]

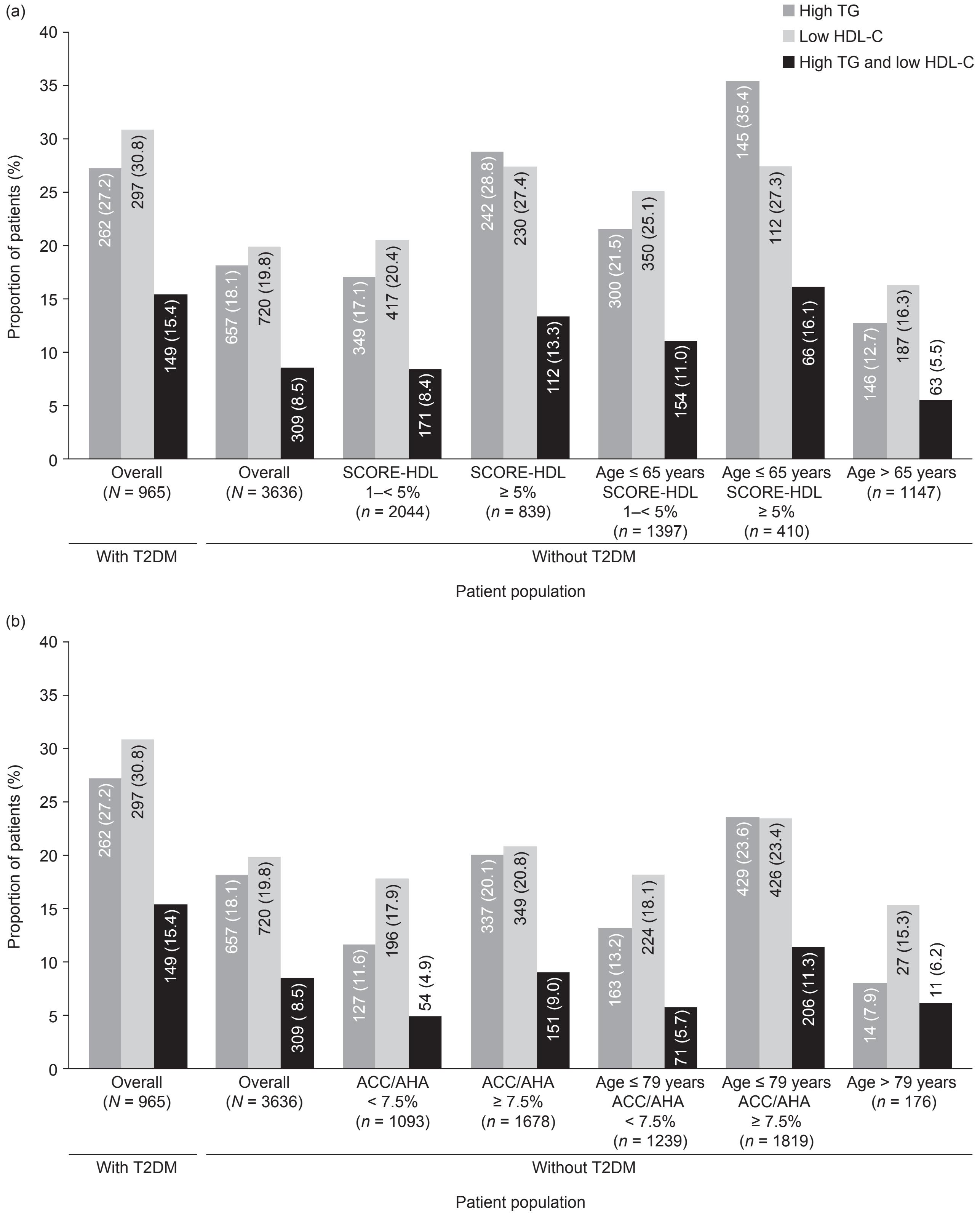

Figure S1 Proportion of non-statin-treated patients with markers of atherogenic dyslipidemia, according to T2DM status, CVD risk, and age

(a) According to SCORE-HDL categories; (b) According to ACC/AHA categories. Data within bars are n (%). High TG: ≥ 2.3 mmol/l. Low HDL-C: < 1.0 mmol/l in men and < 1.3 mmol/l in women. *Abbreviations: ACC* American College of Cardiology, *AHA* American Heart Association, *CVD* cardiovascular disease, *HDL-C* high-density lipoprotein cholesterol, *SCORE-HDL* Systematic Coronary Risk Evaluation-high-density lipoprotein, *T2DM* type 2 diabetes mellitus, *TG* triglycerides
